# Major Gene with Polygene Inheritance Analysis of Prostrate Growth Habit in Hybrids of *Chrysanthemum yantaiense* × *C. indicum*

**DOI:** 10.3390/plants14091338

**Published:** 2025-04-29

**Authors:** Dawei Li, Yuxian Xu, Yuchao Tang, Tongjun Zhou, Hai Li, Ziyu Guo, Yilin Liang, Yuxin Wang, Yuyuan Chen, Ming Sun

**Affiliations:** State Key Laboratory of Efficient Production of Forest Resources, Beijing Key Laboratory of Ornamental Plants Germplasm Innovation and Molecular Breeding, National Engineering Research Center for Floriculture, Beijing Laboratory of Urban and Rural Ecological Environment, Key Laboratory of Genetics and Breeding in Forest Trees and Ornamental Plants of Ministry of Education, School of Landscape Architecture, Beijing Forestry University, Beijing 100083, China; lidawei929@bjfu.edu.cn (D.L.); xuyuxian@bjfu.edu.cn (Y.X.); tangyuchao@bjfu.edu.cn (Y.T.); zhoutj000@bjfu.edu.cn (T.Z.); lihai@bjfu.edu.cn (H.L.); gzybfu@bjfu.edu.cn (Z.G.); elaineleung@bjfu.edu.cn (Y.L.); jojo3210398@bjfu.edu.cn (Y.W.); yuyuan2024@bjfu.edu.cn (Y.C.)

**Keywords:** prostrate chrysanthemum, wild germplasm, plant architecture, inheritance, joint segregation analysis

## Abstract

Plant architecture is a crucial trait for ornamental plants. Chrysanthemum with prostrate growth habit is a novel cultivar group of ground-cover chrysanthemum, which have high ornamental value, strong lodging resistance, and outstanding landscape greening capability. To explore the genetic mechanism underlying the prostrate growth habit in chrysanthemum, we used tetraploid prostrate-type *Chrysanthemum yantaiense* as the female parent and erect-type *Chrysanthemum indicum* as the male parent to produce four generations (P_1_, P_2_, F_1_, F_2_). Five traits related to prostrate growth habit in chrysanthemum were investigated including plant height (PH), crown width of the plant (CP), creeping index (CI), gravitropic set-point angle (GSA), and growth habit (GH). The major gene plus polygene mixed inheritance analysis was conducted on five traits across four generations over two years. For the five traits, the coefficients of variation (CVs) were wide-ranging and high (16.64–42.75%), with the PH having the highest CV among them. Genetic analysis revealed that PH conformed to the additive-dominant-epistatic polygene model (C-0) and the model of two equally dominant major genes plus additive-dominant polygene (E-5). The most suitable genetic model for CI was an additive-dominant major gene plus additive-dominant-epistatic polygene model (D-0). The best-fit models for CP and GH were both C-0. For GSA, the best-fit models were E-4 and C-0. Additionally, it appeared that both genetic and environmental factors influenced the prostrate growth habit, as the heritability of major genes and polygenes was less than 50%. This study can serve as a theoretical foundation for the mapping of quantitative trait loci (QTLs) and further exploration of the genetic mechanisms underlying plant architecture in chrysanthemum.

## 1. Introduction

Chrysanthemum (*Chrysanthemum morifolium*) is a significant ornamental crop with a substantial market share in the global flower industry [1]. Plant architecture plays an important role in consumer choice during the commercialization of ornamental plants [2]. Ground-cover chrysanthemum is a new cultivar group characterized by low plant architecture or prostrate growth habit, high ornamental value, robust lodging resistance, and excellent ground coverage capacity [3]. These features also provide high ecological functionality and great potential for landscaping applications. For these reasons, prostrate growth habit has become a crucial breeding goal for chrysanthemum.

The various plant architectures are a result of prolonged evolutionary processes and natural selection, involving intricate genetic and environmental interactions [4]. Research on plant architecture is a frontier and global challenge in plant science [5]. Studies of plant architecture have mainly concentrated on field crops, which can significantly impact crop yield. Breeding semi-dwarf and lodging-resistant varieties remarkably increased wheat and rice yields [6]. In crape myrtle, the joint segregation analysis was employed to investigate the inheritance of plant height (PH), internode length (IL), and primary lateral branch height (PLBH) in six generations [7]. In *Viola cornuta*, seven shoot architecture traits were analyzed using a major gene plus polygene mixed inheritance model in four generations, and PH was found to be regulated by an additive major gene, while the other traits were controlled by two additive major genes [8]. The findings from the genetic study of weeping traits in apple trees suggested that weeping was controlled by a major dominant gene located on chromosome 13 and modified by polygenes [9]. Previous genetic studies on chrysanthemum have focused on quantitative trait loci (QTLs) related to floral traits [10,11,12], research on the prostrate growth habit of chrysanthemum was rare, and its genetic mechanism remains unclear.

Cultivated chrysanthemums are characterized by high levels of heterozygosity and polyploidy, accompanied by complex genetic background and large genome, posing challenges for molecular biological research [13]. Nevertheless, the hybrid offspring of chrysanthemums exhibit a wide range of phenotypic variation, enabling the development of new cultivars with desirable traits through continuous artificial hybridization and selection. Traditional cross-breeding remains a classic and efficient breeding method for chrysanthemum. Wild chrysanthemum, as the primary progenitors of cultivated chrysanthemum, has introduced diverse germplasm resources into chrysanthemum breeding [14]. In the 1940s, the British Sutton Seed Company developed the Cliff Chrysanthemum by hybridizing chrysanthemum with wild chrysanthemum [15]. Ground-cover chrysanthemum cultivars were created through the traditional hybridization of wild chrysanthemums, such as *Chrysanthemum makinoi* and *Chrysanthemum indicum*, with American cultivated varieties, and this innovative approach yielded new cultivars characterized by low plant architecture, abundant flowers, and robust resistance [3]. Cultivated chrysanthemum was hybridized with wild species, such as *Chrysanthemum boreale* and *Chrysanthemum japonicum* from Japan, producing new cultivars with early flowering and diverse flower colors [16]. It has been reported that there is an attempt to crossbreed wild chrysanthemum with cultivated varieties in order to incorporate the superior drought-resistance traits of wild chrysanthemum into cultivated chrysanthemum [17]. These efforts have greatly advanced the breeding process of chrysanthemum.

Genetic analysis of quantitative traits is crucial as it provides essential direction and theoretical basis for more efficient breeding. A major gene plus polygene mixed inheritance model has been widely used in plant genetics research in recent years. Compared to previous methods of genetic analysis, this model not only detects and identifies major genes and polygenes but also estimates genetic parameters such as gene effects and heritability [18,19], and it is more efficient and cost-effective than methods like QTL and GWAS, which require substantial financial investment for the acquisition of molecular data. Research has shown that joint segregation analysis and QTL mapping can serve as mutual verification and supplementation [20]. This genetic model has been broadly applied in studies of diverse crops, such as internode length in tomatoes [21], vitamin P content in eggplants [22], and ornamental traits in irises [23,24].

Establishing identification methods and evaluation criteria for prostrate chrysanthemum is necessary before conducting genetic analysis. The prostrate growth habit was characterized by low plant height, wide crown width of the plant, and large branch angle. Currently, identification methods and evaluation criteria for chrysanthemum plant architecture primarily include plant height, plant width, and primary branch length [25,26]. The plant architecture of prostrate chrysanthemum differs significantly from that of cut chrysanthemum, and these standards and methods may not be appropriate for prostrate chrysanthemum. Plant growth habits directly affect the morphology of plants, such as the number of branches and the growth posture (erect, prostrate, intermediate), which determines the cultivation, maintenance, and application of plants [27]; genetic studies of plant growth habit have been carried out in many plants [28,29,30]. Gravity has an extremely important effect on plant architecture. The lateral branches of higher plants usually maintain an angle to gravity, and this angle is called the gravitropic set-point angle (GSA) [31]. Research on the GSA of prostrating mutants in rice found that the *LAZY1* gene leads to a prostrate phenotype [32].

There have been few reports on the genetic analysis and standard evaluation and identification methods for prostrate chrysanthemum, which seriously inhibited the breeding process. In the present study, we used four generations (P_1_, P_2_, F_1_, F_2_) derived from a cross between parents with contrasting plant architecture. Additional indicators, including CI, GSA, and GH, along with the traditional plant architecture indicators PH and CP, were evaluated and identified. Utilizing a major gene plus polygene mixed inheritance model, this study evaluated the genetic composition of plant architecture traits and estimated the genetic effects of the genes. The findings are expected to provide a theoretical basis for corresponding breeding strategies and lay a foundation to explore the genetic mechanism of prostrate traits in chrysanthemum.

## 2. Results

### 2.1. Frequency Distributions and Statistical Analysis

In the progeny populations, the means of all traits fell between the two parental lines, whereas the extremums of CI and GSA exceeded that of the parental lines (Table 1). The coefficient of variation (CV) for the five traits among the progeny ranged from 16.64% to 42.75%. The PH and CI showed extensive variability in chrysanthemum plant architecture. In the F_1_ population, PH exhibited the highest CV (42.75% in 2021), followed by CI (41.31% in 2021). In the F_2_ population, CI had the highest CV (34.75% in 2021), followed by PH (33.84% in 2022). The coefficients of variation for GH, CP, and GSA all exceeded 16% (16.64–35.17%), indicating that the progenies had higher genetic variation.

**Figure 1:**
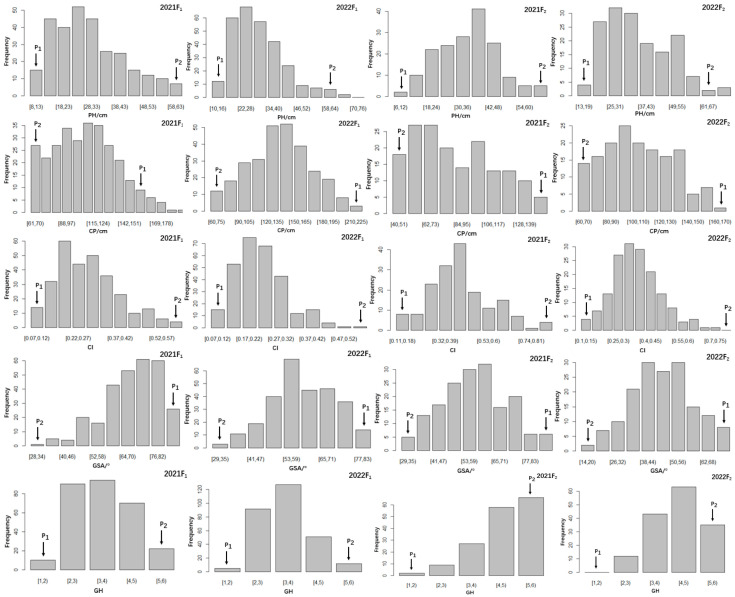
Frequency distribution of the five traits in F_1_ and F_2_ populations. PH, plant height; CP, crown width of the plant; CI, plant height/crown width of the plant; GSA, gravitropic set-point angle; GH, growth habit.

The frequency distributions of the five traits over two years in the segregating populations are illustrated in Figure 1. According to skewness and kurtosis (Table 1), five plant architecture traits exhibited continuous distribution aligning with a normal distribution or a partial distribution with varying degrees in the F_1_ and F_2_ populations, implying that all traits have distinct quantitative genetic characteristics.

### 2.2. Correlation Between Plant Architecture Traits

**Figure 2:**
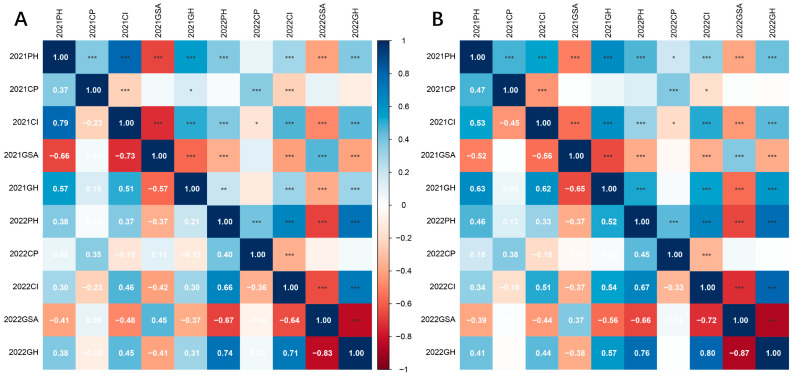
Pearson’s correlation matrix among plant architecture traits in populations. (**A**) correlation analysis results of the five traits in F_1_. (**B**) correlation analysis results of the five traits in F_2_. PH, plant height; CP, crown width of the plant; CI, plant height/crown width of the plant; GSA, gravitropic set-point angle; GH, growth habit * *p* < 0.05; ** *p* < 0.01; *** *p* < 0.001.

Correlation analysis demonstrated that the correlations among the five traits were similar in the segregating populations in both years (Figure 2). In both 2021 and 2022, the correlation between PH and CI exhibited significantly positive (0.79, 0.66 in F_1_, 0.53, 0.67 in F_2_), indicating that lower plant height was associated with a smaller creeping index. Additionally, GH showed a significant positive correlation with PH and CI in 2022 (0.74, 0.71 in F_1_, 0.76, 0.80 in F_2_). GSA was found to be significantly and negatively correlated with GH, PH, and CI in both years, with correlation coefficients ranging from −0.57 to −0.83 in F_1_, and −0.52 to −0.87 in F_2_, the result revealed that a larger GSA corresponds to a stronger prostrate growth habit. Furthermore, significant correlations were observed between the same traits across different years, suggesting that the identification of plant architecture phenotypes was accurate and stable.

### 2.3. Evaluation of Optimal Genetic Models

The optimal models were selected based on the Akaike information criterion (AIC) value (Table 2). All candidate models were tested for goodness of fit, including the Uniformity test (U_1_^2^, U_2_^2^, U_3_^2^), the Smirnov test (_n_W^2^), and the Kolmogorov test (D_n_), to determine the optimal model (Table 3). Taking the PH as an example, the models C-0 and E-4 in 2021, and E-6 and E-5 in 2022, which had the smallest or the second minimum AIC values, were selected as candidate optimal models for the goodness of fit tests. It was found that the number of values below the statistical significance threshold for models C-0 (2021), E-4 (2021), E-6 (2022), and E-5 (2022) were 2, 5, 5, and 4, respectively. Combining the goodness of fit test results with the AIC values, we finally determined that models C-0 and E-5 were the optimal models for PH in 2021 and 2022, respectively. The results showed that the inheritance of PH conforms to the additive-dominance-epistasis polygene model (C-0) and the two equally dominant major genes plus the additive-dominance polygene model (E-5).

Similarly, the optimal model for CP and GH was identified as C-0 in both years, implying that the inheritance of CP and GH conforms to the additive-dominance-epistasis polygene model (C-0). For the CI trait, the best-fit model in both years was determined to be D-0, which corresponds to one additive-dominance major gene plus the additive-dominance-epistasis polygene model. The optimal model for GSA in 2021 was E-4, determined by two equally additive-effect major genes and additive-dominance polygene, while the most suitable model in 2022 was C-0.

### 2.4. Genetic Parameter Estimation of Plant Architecture Traits Under the Optimal Genetic Model

The first-order parameters of the optimal genetic models for the five traits were observed (Table 4). The PH trait conformed to model E-5 in 2022. Specifically, two pairs of major genes and polygenes showed additive and dominant effects. The additive effect of the first major gene was −1.3315 and the second major gene exhibited an additive effect of −9.1163, which were less than that of the polygene (−13.484). Additionally, the dominance effect of the polygene was 0.5898 which was also lower than the additive effects (−13.484). The CI fitted model D-0 in both years, with major genes exhibiting an additive-dominant effect. In 2021, the additive effect of the major gene was −0.1219, and the dominance effect was −0.1217, suggesting the dominance effect was less than the additive effect. In 2022, the additive effect of the one major gene was −0.0839, and the dominance effect was −0.0836. For GSA was consistent with the model E-4 in 2021. The additive effect of the first major gene was 6.3498, with polygenes having an additive effect of 11.1966 and a dominance effect of 17.7547, highlighting that the dominance effect of polygene was the largest in all gene effects, and dominant effects are closely related to heterosis in hybrid generations.

Second-order genetic parameters of the optimal models for the five traits are listed in (Table 4). In 2022, the heritability of the major gene for PH was 29.39%, with polygenic heritability being 0%. For CP, the polygenic variance and polygenic heritability were 78.1598 and 9.40% in 2021, respectively. The heritability of the major gene for CI was found to be 41.57% and 39.48%, with polygene heritability values of 0% and 5.45% in 2021 and 2022, respectively, indicating that CI was mainly controlled by major genes and slightly modified by polygenes. In 2021, the major gene heritability of GSA was 23.12%, while the polygenic heritability was 0%. In 2022, GSA was mainly regulated by polygenes, with polygenic heritability being 26.69%. The heritability of polygenes for CP and GH ranged from 0% for CP to 9.40% for GH in 2021, and from 0% for CP to 28.19% for GH in 2022. The heritability of genes associated with these traits remained below 50%, suggesting that selection for these traits should be deferred to late generations.

## 3. Discussion

Plant architecture is vital in chrysanthemum breeding, providing substantial ornamental, economic, and ecological benefits [33]. The prostrate growth habit effectively addresses the issue of lodging in chrysanthemum cultivation and application, introducing innovative varieties of chrysanthemum to consumers and enhancing the ground cover capacity in landscaping. The genetic mechanisms regulating quantitative traits are complex. In species such as chrysanthemum, which possess an enormous and complex genome, the major gene plus polygene mixed inheritance analysis is an alternative method for identifying QTLs with significant genetic effect. Currently, most of the genetic analysis on chrysanthemum have focused on the floral traits [25,34,35], whereas few studies have focused on the inheritance of prostrate growth habit. Moreover, previous studies have relied on single-generation segregation analysis in chrysanthemum [34,35,36], which has limitations in detecting the polygenic effects. In this study, four generations were derived from two parents with contrasting plant architecture, and joint segregation analysis was used to investigate the inheritability and gene effects of plant architecture in prostrate chrysanthemum. The prostrate growth habit of *Chrysanthemum yantaiense* is a complex quantitative trait that exhibits significant differences in plant architecture compared to cultivated chrysanthemum (*Chrysanthemum morifolium*). Current single-parameter evaluation methods are insufficient for its accurate assessment. Previous studies have demonstrated that multidimensional phenotypic analysis enables more comprehensive characterization of complex traits and provides better resolution of their underlying genetic mechanisms [37]. In this study, we integrated and optimized previously established evaluation criteria for prostrate growth habit, combined with field phenotypic observations. The trait was divided into five components—plant height (PH), crown width of the plant (CP), creeping index (CI), gravitropic set-point angle (GSA), and growth habit (GH)—which were used collectively to assess the prostrate growth habit of chrysanthemum. Correlation analysis and coefficients of variation among these traits indicated that this evaluation system can effectively assess prostrate growth habit and its variation in the progeny.

The genetic analysis using joint segregation analysis revealed that the inheritance of PH in 2021 conforms to an additive-dominant-epistatic polygene model (C-0), indicating that PH may be controlled by polygenes. This finding aligns with previous studies on plant architecture that employed single-generation analyses in chrysanthemum [26]. In 2022, the result of genetic analysis illustrated that PH was not only influenced by polygenes but also controlled by two pairs of major genes with equally dominant effect. The additive effect of the polygenes was greater than two pairs of major genes, and the dominance effect of the polygenes was very low; these results implied that PH may be influenced by multiple genes with the additive effect. The additive gene effect was stable and controllable, making the selection of traits governed by additive gene effects highly effective. The genetic contribution of the genes was less than 50%, suggesting the significant influence of environmental factors on PH. Our results were consistent with the findings of PH which used six generations of crape myrtle [7], proposing that the selection of this trait should consider environmental factors and be conducted in late generations. The genetic analysis results for PH were also consistent with the QTL mapping findings in crape myrtle [38] and the study of GWAS in chrysanthemum [33] indicating that PH may be controlled by multiple genes and vulnerable to environmental influences.

The research findings highlighted that the genetic model for CI was D-0, which conforms to an additive-dominant major gene plus additive-dominant-epistatic polygene model. The additive effects of the major gene were similar to the dominant effects, implying that the additive and dominant effect influenced this character equally. The genetic contribution of the major genes was the largest in all traits, while the polygenic contributions were relatively small. These results exhibited that the substantial variation in CI was controlled by major genes with minor modifications by polygenes. These findings demonstrated that CI was influenced by a combination of genetic and environmental factors, implying that selection should be performed in late generations. Our study elucidated that the GSA was described by the E-4 model in 2021, controlled by two pairs of major genes plus additive-dominant polygene. Previous forward genetics studies also revealed that the branching angle of prostrate habits in rice may be associated with two genes involved in gravity response regulation, namely *LAZY1* and *PROG1* [32,39]. Additionally, we found that the dominant effect of polygenes was the strongest among all effects, and the model in 2022 conformed to C-0. We speculated that, in addition to the two aforementioned genes regulating GSA in rice, there exist other genetic factors controlling GSA in chrysanthemum, a notion that has also been supported by our subsequent research (unpublished results). The GH and CP both fitted the C-0 genetic model, indicating that GH and CP were not controlled by major genes but were primarily influenced by polygenes. This finding implied that the polygenic effects of GH may contribute substantially, consistent with the identification of multiple QTLs associated with growth habit(GH) across various linkage groups in peanuts [40]. The heritability estimates for GH were below 30%, implying a predominant influence of environmental factors. For CP, the heritability of polygene ranged from 0% to 9.40%, suggesting that these traits were significantly affected by environmental factors, and it would be helpful to promote this character in an appropriate environment. In summary, we speculate that the prostrate growth habit of *Chrysanthemum yantaiense* may be regulated by polygenes, with the involvement of one or two major genes. Studies on prostrate growth habit in chrysanthemum are rare. In recent years, research on the genetic mechanisms underlying this trait has mainly focused on field crops and economic crops. In a study on peanuts [28], researchers developed a genetic population and constructed a genetic linkage map by crossing prostrate and erect cultivars. Traits related to prostrate growth habit, including LBA, MSH, LBL, ER, and IOPT, were evaluated. Through QTL analysis, a total of 39 QTLs were identified, with 1–2 major QTLs detected for each trait [28]. In wheat, researchers conducted a GWAS analysis on the prostrate/erect growth habit using 184 wheat germplasm accessions, identifying 14 associated loci and two major-effect key loci [29]. In cowpea, plant architecture was classified into three types: prostrate, semi-prostrate, and erect. An association analysis of plant architecture traits was conducted based on SNP markers, and a total of 10 loci were identified [41]. These research findings indicate that the prostrate growth habit is controlled by multiple genetic loci, with the potential presence of 1–2 major-effect loci, which is generally consistent with the genetic analysis results of this study.

By employing phenotype data and joint segregation analysis, we assessed the impact of major genes, polygenes, and their interactions on five plant architecture traits in chrysanthemum. This research provided significant insights for enhancing breeding strategies and laid the groundwork for subsequent molecular investigations. To advance our research further, we plan to conduct genetic profiling of individuals within our population. This will involve constructing a genetic map and integrating phenotype data with QTL mapping to identify the positions of these QTLs on the genome and chromosomes. Based on the data presented, our objective is to develop molecular markers to assist in breeding programs. Additionally, we are utilizing chrysanthemum germplasm described in our paper to produce novelty varieties through traditional hybridization and have selected numerous hybrid lines that showed a prostrate growth habit and superior comprehensive trait. Our goal is to cultivate new varieties characterized by prostrate growth, resistance to lodging, high ornamental value, and outstanding landscape greening capability. These efforts hold significant practical implications for chrysanthemum breeding. This study will lay a solid foundation for the localization of QTLs related to plant architecture in chrysanthemum and the development of molecular markers to aid in breeding programs.

## 4. Materials and Methods

### 4.1. Plant Materials

**Figure 3:**
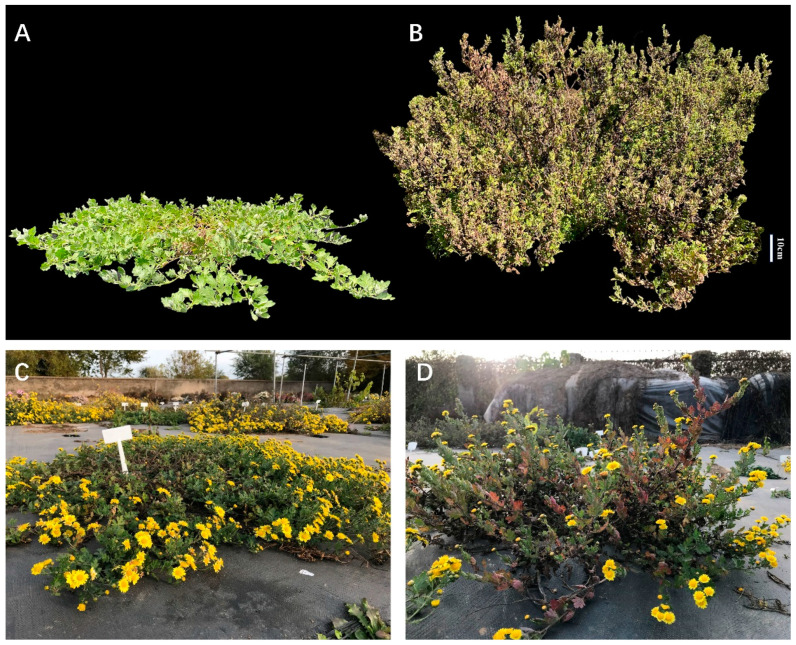
Plant materials used in this study. (**A**) female parent; (**B**) male parent; (**C**,**D**) individual in the F_1_ population.

In this study, four generations (P_1_, P_2_, F_1_, F_2_) were constructed by hybridizing a prostrate wild chrysanthemum (*Chrysanthemum yantaiense*) (Figure 3A), used as the female parent, with an erect wild chrysanthemum (*Chrysanthemum indicum*) (Figure 3B), used as the male parent. The female parent, a wild prostrate chrysanthemum germplasm discovered and named by our research team in the coastal region of Shandong, China [42], and the male parent, a wild chrysanthemum collected from the Shennongjia National Nature Reserve in Hubei Province, are both tetraploid chrysanthemum. In December 2018 and 2019, F_1_ hybrid seeds were obtained through cross-pollination. To prevent contamination from other pollen, flowers were carried out emasculation artificially and were covered with bags after artificial pollination. These seeds were sown in 50-cell plug trays in early March of the following year and transplanted to the chrysanthemum nursery at the China National Engineering Research Center for Floriculture (CNERCF) in Beijing (40°02′ N, 115°50′ E). The cultivation environment and management practices followed conventional field cultivation methods. In October 2019, based on the phenotypic observations of the full-sib F_1_ population in 2019 (obtained in 2018), two F_1_ individuals (F_1_-3 and F_1_-45) with similar phenotypes and both exhibiting intermediate plant architecture were selected. F_1_-3 was used as the female parent and F_1_-45 as the male parent to produce the F_2_ population through hybridization. The methods for the F_2_ generation were the same as those used for the F_1_ generation. The F_1_ and F_2_ populations were cultivated and phenotyped at the same time and under the same experimental conditions. The nursery provided suitable climatic conditions, including appropriate temperature, photoperiod, and soil moisture, ensuring full flowering and development of the plants.

### 4.2. Trait Investigation and Phenotyping

The experiment was conducted in the field during the years 2021 and 2022. Five plant architecture-related traits including plant height (PH), crown width of the plant (CP), creeping index (CI), gravitropic set-point angle of branching (GSA), and growth habit (GH) were evaluated over two years in four generations. A total of 480 individuals were randomly selected for the study, including 293 F_1_ individuals, 171 F_2_ individuals, and eight individuals each from the parent lines. This extensive evaluation was designed to capture the genetic diversity and inheritance patterns of the prostrate growth habit in chrysanthemum.

Field experiments demonstrated that the plant architecture of prostrate chrysanthemum is unique, and conventional chrysanthemum plant architecture indicators are insufficient for accurately identifying this characteristic. For example, two progeny individuals with the same plant height may exhibit significant differences in the crown width of the plant, gravitropic set-point angle, and creeping index, resulting in varying degrees of prostrate growth habit. Except for the classical PH and CP plant architecture indicators, to better describe and evaluate the prostrate growth habit of chrysanthemum, additional indicators such as CI, GSA, and GH were introduced and adjusted according to prostrate chrysanthemum. The phenotypic evaluation methods were as follows:

PH (plant height)—the vertical height of the tallest point of the entire plant, excluding abnormal branches; CP (crown width of the plant)—the average of the longest distances in the east-west and north-south directions of the plant; CI (creeping index)—the ratio of PH to CP (PH/CP) of a plant; GSA (gravitropic set-point angle of branching)—the angle between the secondary branches and the direction of negative gravitropism; GH (growth habit)—the plant architecture was classified into six grades (Grade 1 to 6) based on the branching angles of different levels of shoots (prostrate: 0–30°; semi-prostrate: 30–60°; erect: 60–90°), where Grade 1 represents the strongest prostrate growth habit (completely prostrate), while Grade 6 represents the strongest erect growth habit (completely erect). The phenotypic identification method for GH is detailed in the Table S1.

### 4.3. Statistical and Joint Segregation Analysis

In order to estimate the minimum, maximum, mean, standard deviation, and coefficient of variation for each trait per year, statistical analyses were carried out. Genetic analysis of the four generations employed the major gene plus polygene mixed inheritance model, which categorizes genetic models into five categories, including one major gene model (Model A), two major genes model (Model B), polygene model (Model C), one major gene plus polygene model (Model D), two major genes plus polygene model (Model E) [19]. These genetic models were combined with the phenotypic frequency distributions to obtain the maximum likelihood value (MLV) for each model. The Akaike information criterion (AIC) was used to select the candidate genetic models, with the model having the lowest AIC value being preferred.

To determine the best-fit model, goodness of fit tests were performed using the Uniformity test (U_1_^2^, U_2_^2^, U_3_^2^), Smirnov test (_n_W^2^), and Kolmogorov test (D_n_). The most suitable model was then selected based on these tests. First-order and second-order genetic parameters were estimated using the least-squares method. Finally, the genetic analysis of the four generations was carried out using the SEA software package (Version 2.0.1) [43], which facilitated the joint segregation analysis of the genetic parameters across the multiple generations

## 5. Conclusions

In this study, an evaluation standard for prostrate growth habits of chrysanthemums was established. Genetic analysis revealed that the PH was most likely influenced by multiple genes with the additive effect. The CI was influenced by an additive-dominant major gene plus additive-dominant-epistatic polygene. The GSA may be regulated by multiple genes, and both additive and dominant effects should be considered to function together. The CP and GH were controlled by additive-dominant-epistatic polygenes. The heritability of the genes associated with these traits was below 50%, indicating that these traits should be selected in late generations. The findings of this study provided a foundational basis for QTL mapping and marker-assisted breeding of plant architecture-related traits in Chrysanthemum.

## Figures and Tables

**Table 1:** Descriptive statistics of the plant architecture traits in four generations.

Trait	Year	Generation	Min	Max	Mean	SD	Skewness	Kurtosis	CV (%)
PH/cm	2021	P_1_	12.60	16.60	14.51	1.70	−0.02	−1.96	11.72%
		P_2_	30.70	58.90	42.63	10.16	0.21	−1.59	23.84%
		F_1_	8.10	64.20	29.43	12.58	0.66	−0.20	42.75%
		F_2_	6.30	65.30	34.79	11.56	0.19	−0.11	33.24%
	2022	P_1_	9.20	19.20	11.65	3.56	1.16	−0.31	30.57%
		P_2_	51.70	69.80	59.51	5.51	0.43	−0.90	9.26%
		F_1_	10.20	79.30	30.28	11.16	1.10	1.61	36.84%
		F_2_	13.70	72.50	36.85	12.47	0.56	−0.38	33.84%
CP/cm	2021	P_1_	100.95	155.30	132.63	22.70	−0.20	−1.94	17.12%
		P_2_	39.90	79.20	55.18	15.39	0.55	−1.59	27.89%
		F_1_	61.20	196.90	108.48	27.92	0.42	−0.25	25.74%
		F_2_	40.80	152.60	85.38	28.84	0.43	−0.87	33.78%
	2022	P_1_	174.20	257.90	213.85	33.16	0.28	−1.83	15.51%
		P_2_	77.40	120.80	89.54	14.09	1.19	0.16	15.74%
		F_1_	60.20	231.80	135.30	34.67	0.12	−0.33	25.62%
		F_2_	61.10	175.40	105.34	26.05	0.37	−0.54	24.72%
CI	2021	P_1_	0.10	0.12	0.11	0.01	0.00	−1.98	8.42%
		P_2_	0.62	1.08	0.79	0.15	0.67	−0.74	18.51%
		F_1_	0.08	0.63	0.28	0.11	0.71	0.10	41.31%
		F_2_	0.12	0.88	0.43	0.15	0.55	0.31	34.75%
	2022	P_1_	0.04	0.08	0.06	0.02	0.33	−1.58	27.49%
		P_2_	0.58	0.78	0.67	0.08	0.25	−1.89	11.71%
		F_1_	0.08	0.59	0.23	0.08	0.93	1.46	35.34%
		F_2_	0.11	0.73	0.36	0.11	0.49	0.35	31.70%
GSA/°	2021	P_1_	78.90	82.73	80.73	1.27	−0.08	−1.35	1.58%
		P_2_	18.03	41.97	32.93	7.62	−0.63	−0.81	23.13%
		F_1_	28.10	89.00	68.83	11.46	−0.75	0.33	16.64%
		F_2_	29.30	88.30	58.11	12.85	0.10	−0.43	22.12%
	2022	P_1_	80.90	88.33	84.20	2.66	0.33	−1.67	3.16%
		P_2_	17.83	29.33	25.57	3.91	−0.78	−0.79	15.30%
		F_1_	29.30	87.90	59.75	10.91	−0.13	−0.33	18.27%
		F_2_	14.07	73.13	46.52	12.41	−0.04	−0.40	26.68%
GH\Grade	2021	P_1_	1.00	1.00	1.00	0.00	NA	NA	0.00%
		P_2_	6.00	6.00	6.00	0.00	NA	NA	0.00%
		F_1_	1.00	6.00	3.08	1.08	0.44	−0.25	35.17%
		F_2_	1.00	6.00	4.18	1.01	−0.67	0.24	24.22%
	2022	P_1_	1.00	1.00	1.00	0.00	NA	NA	0.00%
		P_2_	6.00	6.00	6.00	0.00	NA	NA	0.00%
		F_1_	1.00	6.00	2.92	0.87	0.51	0.12	29.88%
		F_2_	2.00	6.00	3.91	1.00	0.06	−0.46	25.54%

PH, plant height; CP, crown width of the plant; CI, plant height/crown width of the plant; GSA, gravitropic set-point angle; GH, growth habit; NA, no data.

**Table 2:** The AIC value of the five traits for various genetic models.

Model Code	Model Implication	AIC Value (2021)					AIC Value (2022)				
		PH	CP	CI	GSA	GH	PH	CP	CI	GSA	GH
A-1	1MG-AD	3784.31	4600.48	−519.46	3776.10	1499.49	3624.61	4651.82	−725.75	3644.90	1321.48
A-2	1MG-A	3786.59	4607.12	−520.15	3783.51	1472.06	3634.58	4637.40	−779.41	3605.72	1287.71
A-3	1MG-EAD	3782.91	4597.52	−516.33	3774.10	1496.05	3626.42	4649.36	−728.11	3642.28	1319.62
A-4	1MG-AEND	3803.68	4651.12	−388.44	3860.11	1572.92	3699.10	4690.91	−581.22	3749.48	1429.92
B-1	2MG-ADI	3779.53	4580.37	−583.68	3746.94	1425.41	3613.99	4574.68	−860.98	3573.78	1251.91
B-2	2MG-AD	3781.62	4592.15	−556.42	3753.61	1466.93	3615.64	4637.49	−828.84	3605.00	1277.94
B-3	2MG-A	3783.62	4609.07	−532.30	3780.51	1463.89	3623.65	4630.79	−819.51	3596.82	1274.22
B-4	2MG-EA	3781.62	4610.08	−534.30	3778.51	1461.89	3621.65	4628.79	−821.51	3594.82	1272.23
B-5	2MG-AED	4091.38	4771.75	−178.96	3993.56	2023.33	4075.73	5305.54	−110.04	4218.27	1888.19
B-6	2MG-EEAD	3777.55	4585.46	−561.96	3748.20	1464.37	3619.24	4634.18	−794.29	3611.66	1285.14
C-0	PG-ADI	**3763.74**	**4570.02**	−597.10	3731.59	**1402.93**	3602.50	**4550.74**	−874.19	**3556.06**	**1232.15**
C-1	PG-AD	3771.96	4582.32	−584.91	3736.88	1433.12	3605.75	4609.24	−847.52	3593.81	1257.06
D-0	MX1-AD-ADI	3767.74	**4571.50**	**−603.22**	3734.93	**1406.93**	3599.48	**4554.74**	**−875.17**	**3559.70**	**1235.54**
D-1	MX1-AD-AD	3774.75	4580.62	−565.46	3738.55	1433.60	3619.28	4607.55	−841.23	3590.81	1255.24
D-2	MX1-A-AD	3773.96	4583.31	−583.01	3737.44	1435.12	3607.24	4611.24	−845.65	3589.61	1254.40
D-3	MX1-EAD-AD	3773.96	4584.31	−582.92	3738.76	1435.12	3607.75	4611.24	−845.53	3593.98	1258.97
D-4	MX1-AEND-AD	3773.96	4579.83	−582.92	3737.55	1435.12	3607.75	4611.24	−845.53	3593.80	1258.97
E-0	MX2-ADI-ADI	3775.74	4579.50	−596.59	3742.76	1412.36	3607.48	4562.74	−869.43	3567.50	1243.30
E-1	MX2-ADI-AD	3771.59	4574.10	**−602.12**	3736.96	1408.19	3601.38	4556.98	**−875.15**	3562.28	1237.58
E-2	MX2-AD-AD	3771.96	4577.83	−591.76	3735.32	1433.12	3598.70	4609.24	−856.12	3587.62	1252.28
E-3	MX2-A-AD	3767.96	4577.34	−589.05	3731.50	1429.12	3601.21	4605.24	−851.71	3583.65	1248.44
E-4	MX2-EA-AD	**3765.96**	4575.55	−591.05	**3729.69**	1427.12	3599.02	4603.24	−853.71	3581.81	1246.57
E-5	MX2-AED-AD	3771.73	4583.16	−593.71	3734.06	1439.16	**3595.99**	4623.43	−858.56	3586.99	1251.78
E-6	MX2-EEAD-AD	3765.96	4576.30	−597.21	**3730.85**	1427.12	**3595.46**	4603.24	−862.12	3585.14	1246.71

PH, plant height; CP, crown width of the plant; CI, plant height/crown width of the plant; GSA, gravitropic set-point angle; GH, growth habit. The AIC values of candidate genetic models are bold.

**Table 3:** Test of goodness of fit for the candidate genetic models.

Trait	Year	Model Code	Generation	U_1_^2^	P(U_1_^2^)	U_2_^2^	P(U_2_^2^)	U_3_^2^	P(U_3_^2^)	_n_W^2^	P(_n_W^2^)	D_n_	P(D_n_)
PH	2021	C-0	P_1_	0.0000	1.0000	0.5843	0.4446	9.3504	**0.0022**	0.4550	0.0533	0.4312	0.0723
			P_2_	0.0027	0.9584	0.0287	0.8655	0.2257	0.6348	0.0332	0.9644	0.0884	1.0000
			F_1_	1.6360	0.2009	0.8573	0.3545	1.5631	0.2112	0.5920	**0.0236**	0.0020	1.0000
			F_2_	0.0268	0.8700	0.1287	0.7198	0.6413	0.4232	0.0758	0.7258	0.0057	1.0000
		E-4	P_1_	5.7686	**0.0163**	6.3987	**0.0114**	0.6662	0.4144	0.9754	**0.0028**	0.6892	**0.0002**
			P_2_	3.7668	0.0523	3.1170	0.0775	0.2068	0.6493	0.3860	0.0822	0.2510	0.6098
			F_1_	3.4167	0.0645	2.1815	0.1397	1.5649	0.2109	0.8163	**0.0067**	0.0024	1.0000
			F_2_	2.1691	0.1408	1.4704	0.2253	0.7287	0.3933	0.2896	0.1514	0.0043	1.0000
	2022	E-6	P_1_	2.8986	0.0887	4.0887	**0.0432**	2.2332	0.1351	0.5777	**0.0257**	0.4046	0.1074
			P_2_	2.6103	0.1062	3.3445	0.0674	1.1188	0.2902	0.3802	0.0853	0.3092	0.3532
			F_1_	3.9417	**0.0471**	4.0172	**0.0450**	0.1075	0.7430	0.7946	**0.0076**	0.0035	1.0000
			F_2_	0.1593	0.6898	0.1190	0.7301	0.0275	0.8682	0.1733	0.3267	0.0032	1.0000
		E-5	P1	2.7050	0.1000	3.9180	**0.0478**	2.3952	0.1217	0.5564	**0.0290**	0.3931	0.1264
			P_2_	2.4277	0.1192	3.1685	0.0751	1.1786	0.2776	0.3584	0.0979	0.2972	0.4005
			F_1_	4.0294	**0.0447**	3.7292	0.0535	0.0025	0.9602	0.7937	**0.0076**	0.0035	1.0000
			F_2_	0.7713	0.3798	0.3329	0.5640	1.1956	0.2742	0.1749	0.3228	0.0035	1.0000
CP	2021	C-0	P_1_	0.0031	0.9558	0.0012	0.9719	0.1266	0.7220	0.0634	0.7965	0.2045	0.8295
			P_2_	0.0031	0.9558	0.2404	0.6239	3.0502	0.0807	0.1296	0.4624	0.1908	0.8834
			F_1_	0.3812	0.5369	0.1135	0.7362	1.0890	0.2967	0.1379	0.4316	0.0028	1.0000
			F_2_	0.4414	0.5064	0.0462	0.8298	2.9363	0.0866	0.3852	0.0825	0.0097	1.0000
		D-0	P_1_	0.0037	0.9516	0.0001	0.9914	0.0368	0.8479	0.0642	0.7918	0.1957	0.8651
			P_2_	0.0038	0.9510	0.2237	0.6362	2.7361	0.0981	0.1222	0.4922	0.1818	0.9134
			F_1_	0.4204	0.5167	0.0244	0.8758	3.5561	0.0593	0.1924	0.2841	0.0030	1.0000
			F_2_	0.2143	0.6434	0.0968	0.7557	0.3007	0.5834	0.2472	0.1974	0.0165	1.0000
	2022	C-0	P_1_	0.0218	0.8827	0.0005	0.9822	0.4370	0.5086	0.0780	0.7138	0.0811	1.0000
			P_2_	0.0035	0.9528	0.4037	0.5252	5.3472	**0.0208**	0.2407	0.2057	0.1606	0.9654
			F_1_	0.0278	0.8676	0.1944	0.6593	5.8057	**0.0160**	0.1358	0.4391	0.0024	1.0000
			F_2_	0.1120	0.7379	0.7945	0.3727	5.1504	**0.0232**	0.1744	0.3239	0.0131	1.0000
		D-0	P_1_	0.0218	0.8827	0.0005	0.9822	0.4370	0.5086	0.0780	0.7138	0.0811	1.0000
			P_2_	0.0035	0.9528	0.4037	0.5252	5.3472	**0.0208**	0.2407	0.2057	0.1606	0.9654
			F_1_	0.0278	0.8676	0.1944	0.6593	5.8057	**0.0160**	0.1358	0.4391	0.0024	1.0000
			F_2_	0.1120	0.7379	0.7945	0.3727	5.1504	**0.0232**	0.1744	0.3239	0.0131	1.0000
CI	2021	D-0	P_1_	0.0019	0.9652	0.3744	0.5406	6.8470	**0.0089**	0.3719	0.0898	0.4099	0.0995
			P_2_	0.1303	0.7181	0.0560	0.8130	0.2038	0.6516	0.0525	0.8616	0.1199	0.9986
			F_1_	0.9557	0.3283	0.9172	0.3382	0.0020	0.9645	0.3842	0.0831	0.0023	1.0000
			F_2_	0.0017	0.9666	0.1242	0.7245	1.5571	0.2121	0.1428	0.4146	0.0030	1.0000
		E-1	P_1_	0.0100	0.9205	0.5695	0.4505	6.9290	**0.0085**	0.3745	0.0884	0.4254	0.0790
			P_2_	0.2081	0.6482	0.1035	0.7477	0.2306	0.6311	0.0619	0.8051	0.1191	0.9987
			F_1_	1.0046	0.3162	1.0223	0.3120	0.0264	0.8710	0.3930	0.0786	0.0023	1.0000
			F_2_	0.0017	0.9669	0.0255	0.8731	0.6395	0.4239	0.1034	0.5785	0.0053	1.0000
	2022	D-0	P_1_	0.0000	1.0000	0.6250	0.4292	10.0000	**0.0016**	0.6667	**0.0154**	0.5000	**0.0227**
			P_2_	0.0118	0.9133	0.0020	0.9647	0.0596	0.8071	0.0782	0.7127	0.0961	1.0000
			F_1_	0.6038	0.4371	1.3091	0.2526	2.4560	0.1171	0.3166	0.1278	0.0035	1.0000
			F_2_	0.0078	0.9297	0.0522	0.8192	0.3277	0.5670	0.0258	0.9878	0.0053	1.0000
		E-1	P_1_	0.0287	0.8656	0.9057	0.3413	9.9285	**0.0016**	0.6691	**0.0152**	0.5173	**0.0164**
			P_2_	0.0516	0.8202	0.0206	0.8860	0.0939	0.7592	0.0840	0.6807	0.1027	0.9999
			F_1_	0.6791	0.4099	1.3596	0.2436	2.1680	0.1409	0.3196	0.1254	0.0035	1.0000
			F_2_	0.0051	0.9429	0.0002	0.9896	0.0506	0.8220	0.0125	0.9999	0.0043	1.0000
GSA	2021	E-4	P_1_	4.0049	**0.0454**	2.4196	0.1198	2.3369	0.1263	0.8483	**0.0056**	0.2364	0.6810
			P_2_	3.1866	0.0742	2.7994	0.0943	0.0489	0.8250	0.3960	0.0771	0.0899	1.0000
			F_1_	2.8705	0.0902	3.0203	0.0822	0.1520	0.6967	0.6285	**0.0192**	0.0345	0.8652
			F_2_	1.4888	0.2224	1.2374	0.2660	0.0762	0.7825	0.1655	0.3465	0.0122	1.0000
		E-6	P_1_	4.0217	**0.0449**	2.4372	0.1185	2.3175	0.1279	0.8500	**0.0056**	0.2362	0.6824
			P_2_	3.2014	0.0736	2.8127	0.0935	0.0490	0.8249	0.3977	0.0763	0.0900	1.0000
			F_1_	2.8635	0.0906	2.9772	0.0844	0.1211	0.7279	0.6275	**0.0193**	0.0347	0.8593
			F_2_	2.4481	0.1177	2.0042	0.1569	0.1576	0.6914	0.2606	0.1815	0.0130	1.0000
	2022	C-0	P_1_	0.0000	0.9974	0.5088	0.4757	8.0682	**0.0045**	0.3226	0.1230	0.3488	0.2249
			P_2_	0.0005	0.9815	0.3689	0.5436	6.3474	**0.0118**	0.2402	0.2063	0.3616	0.1917
			F_1_	0.0147	0.9034	0.1706	0.6796	1.3976	0.2371	0.1044	0.5735	0.0040	1.0000
			F_2_	0.0005	0.9826	0.0047	0.9453	0.1287	0.7198	0.0288	0.9799	0.0157	1.0000
		D-0	P_1_	0.0000	0.9974	0.5088	0.4757	8.0682	**0.0045**	0.3226	0.1230	0.3488	0.2249
			P_2_	0.0005	0.9815	0.3689	0.5436	6.3474	**0.0118**	0.2402	0.2063	0.3616	0.1917
			F_1_	0.0147	0.9034	0.1706	0.6796	1.3976	0.2371	0.1044	0.5735	0.0040	1.0000
			F_2_	0.0005	0.9826	0.0047	0.9453	0.1287	0.7198	0.0288	0.9799	0.0157	1.0000
GH	2021	C-0	P_1_	0.0000	1.0000	0.6250	0.4292	10.0000	**0.0016**	0.6667	**0.0154**	0.5000	**0.0227**
			P_2_	0.0000	1.0000	0.6250	0.4292	10.0000	**0.0016**	0.6667	**0.0154**	0.5000	**0.0227**
			F_1_	0.5813	0.4458	0.0862	0.7691	3.1638	0.0753	2.1808	**0.0000**	0.0024	1.0000
			F_2_	0.6457	0.4217	0.4264	0.5137	0.2500	0.6171	1.5860	**0.0001**	0.0398	0.9410
		D-0	P_1_	0.0000	1.0000	0.6250	0.4292	10.0000	**0.0016**	0.6667	**0.0154**	0.5000	**0.0227**
			P_2_	0.0000	1.0000	0.6250	0.4292	10.0000	**0.0016**	0.6667	**0.0154**	0.5000	**0.0227**
			F_1_	0.5814	0.4458	0.0860	0.7693	3.1676	0.0751	2.1810	**0.0000**	0.0024	1.0000
			F_2_	0.6455	0.4217	0.4258	0.5140	0.2513	0.6161	1.5860	**0.0001**	0.0398	0.9408
	2022	C-0	P_1_	0.0000	1.0000	0.6250	0.4292	10.0000	**0.0016**	0.6667	**0.0154**	0.5000	**0.0227**
			P_2_	0.0000	1.0000	0.6250	0.4292	10.0000	**0.0016**	0.6667	**0.0154**	0.5000	**0.0227**
			F_1_	0.6114	0.4342	0.3949	0.5298	0.2651	0.6066	3.2069	**0.0002**	0.0033	1.0000
			F_2_	0.0049	0.9443	0.0007	0.9792	0.1406	0.7077	1.2477	**0.0007**	0.0181	1.0000
		D-0	P_1_	0.0000	1.0000	0.6250	0.4292	10.0000	**0.0016**	0.6667	**0.0154**	0.5000	**0.0227**
			P_2_	0.0000	1.0000	0.6250	0.4292	10.0000	**0.0016**	0.6667	**0.0154**	0.5000	**0.0227**
			F_1_	0.6176	0.4319	0.3766	0.5394	0.3471	0.5558	3.2151	**0.0002**	0.0033	1.0000
			F_2_	0.0003	0.9863	0.0050	0.9439	0.0463	0.8297	1.2371	**0.0007**	0.0200	1.0000

PH, plant height; CP, crown width of the plant; CI, plant height/crown width of the plant; GSA, gravitropic set-point angle; GH, growth habit. The bolded values represent significance at the 0.05 level.

**Table 4:** The first-order and second-order genetic parameters estimation of the optimal model for the five traits.

Trait	Year	Model	1st Order Genetic Parameter						2nd Order Genetic Parameter			
			d_a_	d_b_	h_a_	h_b_	[d]	[h]	σ^2^_mg_	h^2^_mg_ (%)	σ^2^_pg_	h^2^_pg_
PH	2021	C-0	–	–	–	–	–	–	–	–	0.00	0.00%
	2022	E-5	−1.3315	−9.1163	–	–	−13.484	0.5898	45.6899	29.39%	0.00	0.00%
CP	2021	C-0	–	–	–	–	–	–	–	–	78.1598	9.40%
	2022	C-0	–	–	–	–	–	–	–	–	0.00	0.00%
CI	2021	D-0	−0.1219	–	−0.1217	–	–	–	0.0094	41.57%	0.00	0.00%
	2022	D-0	−0.0839	–	−0.0836	–	–	–	0.0051	39.48%	0.0007	5.45%
GSA	2021	E-4	6.3498	–	–	–	11.1966	17.7547	38.1995	23.12%	0.00	0.00%
	2022	C-0	–	–	–	–	–	–	–	–	41.1057	26.69%
GH	2021	C-0	–	–	–	–	–	–	–	–	0.00	0.00%
	2022	C-0	–	–	–	–	–	–	–	–	0.2815	28.19%

d_a_, additive effect of the first major gene; d_b_, additive effect of the second major gene; h_a_, dominant effect of the first major gene; h_b_, dominant effect of the second major gene; [d], additive effect of polygene; [h], dominant effect of polygene; σ^2^_mg_, major gene variance; h^2^_mg_ (%), major gene heritability; σ^2^_pg_, polygene variance; h^2^_pg_ (%), polygene heritability; ‘–’, no data. PH, plant height; CP, crown width of the plant; CI, plant height/crown width of the plant; GSA, gravitropic set-point angle; GH, growth habit.

## Data Availability

All data generated by this research can be found in the main text and Supplementary Materials. Further questions may be addressed to the corresponding author.

## Supplementary Materials

The following supporting information can be downloaded at: https://www.mdpi.com/article/10.3390/plants14091338/s1, Table S1: Standard growth habit grading.
