# Supplementary material for: Major Gene with Polygene Inheritance Analysis of Prostrate Growth Habit in Hybrids of Chrysanthemum yantaiense × C. indicum
Source: Plants (Basel). 2025 Apr 29;14(9):1338. doi: 10.3390/plants14091338 (PMC12073599; doi:10.3390/plants14091338)
Supplement: Supplementary file 1 [file plants-14-01338-s001.zip › plants-3581780-supplementary.pdf]

Table S1

Standard growth habit grading.

Grade	Primary branch	Secondary branch	Inflorescence
I	Prostrate	Prostrate	Prostrate
II	Prostrate	Prostrate	Semi-prostrate
III	Prostrate	Prostrate	Erect
IV	Prostrate	Semi-prostrate	Erect
V	Semi-prostrate	Erect	Erect
VI	Erect	Erect	Erect
